# The first possible choristoderan trackway from the Lower Cretaceous Daegu Formation of South Korea and its implications on choristoderan locomotion

**DOI:** 10.1038/s41598-020-71384-1

**Published:** 2020-09-02

**Authors:** Yuong-Nam Lee, Dal-Yong Kong, Seung-Ho Jung

**Affiliations:** 1 School of Earth and Environmental Sciences, Seoul National University, Seoul, 08826 Republic of Korea; 2 National Research Institute of Cultural Heritage, Daejeon, 35204 Republic of Korea

**Keywords:** Evolution, Palaeontology

## Abstract

Here we report a new quadrupedal trackway found in the Lower Cretaceous Daegu Formation (Albian) in the vicinity of Ulsan Metropolitan City, South Korea, in 2018. A total of nine manus-pes imprints show a strong heteropodous quadrupedal trackway (length ratio is 1:3.36). Both manus and pes tracks are pentadactyl with claw marks. The manus prints rotate distinctly outward while the pes prints are nearly parallel to the direction of travel. The functional axis in manus and pes imprints suggests that the trackmaker moved along the medial side during the stroke progressions (entaxonic), indicating weight support on the inner side of the limbs. There is an indication of webbing between the pedal digits. These new tracks are assigned to *Novapes ulsanensis*, n. ichnogen., n. ichnosp., which are well-matched not only with foot skeletons and body size of *Monjurosuchus* but also the fossil record of choristoderes in East Asia, thereby *N. ulsanensis* could be made by a monjurosuchid-like choristoderan and represent the first possible choristoderan trackway from Asia. *N. ulsanensis* also suggests that semi-aquatic choristoderans were capable of walking semi-erect when moving on the ground with a similar locomotion pattern to that of crocodilians on land.

## Introduction

**Figure 1:**
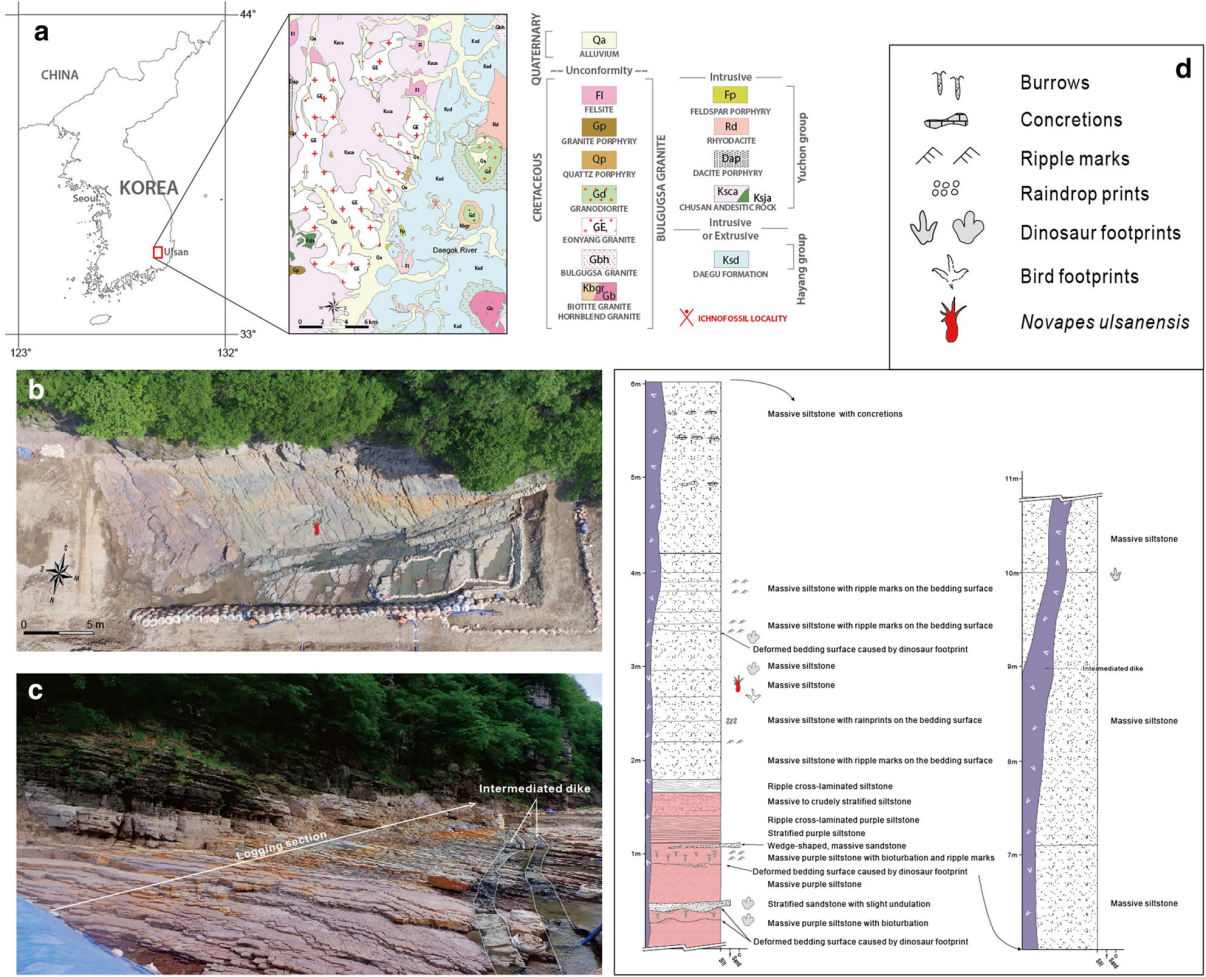
Bangudae tracksite in Ulsan Metropolitan City, South Korea. (**a**) Geographic location and geologic map in the vicinity of the tracksite in the South Gyeongsang Province, South Korea (35°36.255′N, 129°10.706′E). Modified from Lee and Lee^49^. (**b**) Aerial photograph of the tracksite. The red symbol indicates the location of *Novapes ulsanensis*. (**c**) Field photography of Bangudae tracksite, showing the logging section and intermediated dikes. (**d**) Stratigraphic section of the tracksite with the legend. Adobe Illustrator CC (version 24.0.1, https://www.adobe.com/kr/products/illustrator.html) was employed to produce Fig. 1a,d.

South Korea has become globally famous for various tetrapod footprints from Cretaceous strata^1^, among which some clades such as frogs^2^, birds^3^ and mammals^4^ have been proved for their existences only with ichnological evidence. Recently, a new quadrupedal trackway with a pronounced heteropody (sensu stricto)^5^ was discovered in the Daegu Formation (Albian, Lower Cretaceous), Ulsan City, which consists of nine manus-pes sets (Fig. 1). Both manus and pes are pentadactyl with sharp claw marks. They look different from any known quadrupedal dinosaur footprints because there is a clear indication of webbing between the pedal digits. During the Mesozoic, the webbed footprints have been occasionally reported in various quadrupedal animals such as freshwater turtles^6^, amphibians^7^, crocodyliforms^8^, and pterosaurs^9^. However, new footprints differ from their tracks in morphology and appear to belong to that of other clade. Therefore, the purpose of this paper is to describe the new type of tracks and to investigate the possible trackmaker by comparing their ichnological features with skeletal ones. In addition, the trackmaker’s locomotory posture on land was inferred from the trackway pattern.

## Results

### Systematic ichnology

**Figure 2:**
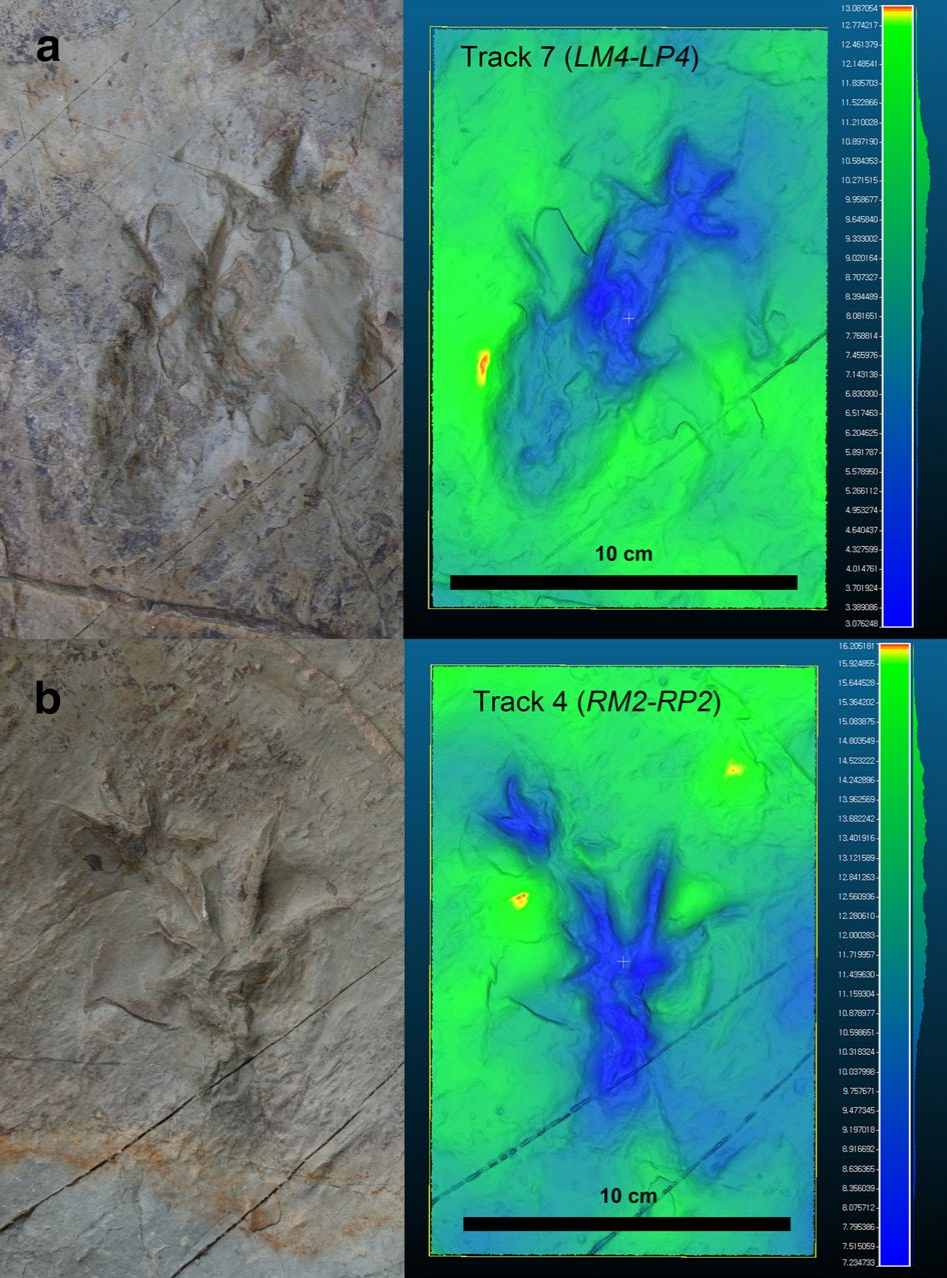
*Novapes ulsanensis*, n. ichnogen., n. ichnosp. (**a**) True-colour and ‘false colour’ images of Track 7 (LM4-LP4). (**b**) True-colour and ‘false colour’ images of Track 4 (RM2-RP2). CloudCompare (version 2.10.1, https://www.danielgm.net/cc/) was employed to produce ‘false colour’ images with relative depth legend in meters.

**Figure 3:**
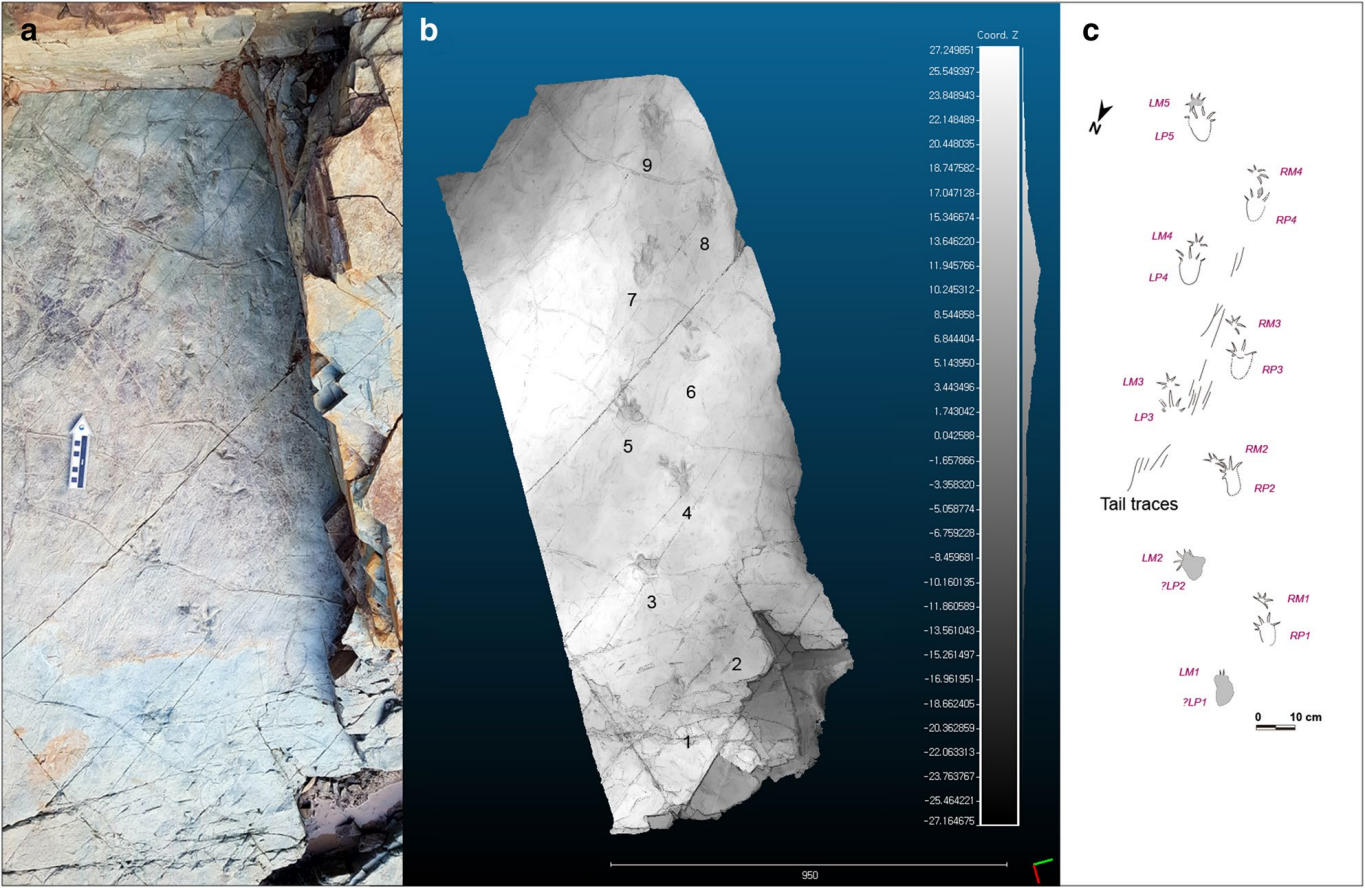
*Novapes ulsanensis* at Bangudae tracksite. (**a**) Field photography in situ before the excavation. (**b**) Grey colour depth map of the trackway with relative depth legend in meters using CloudCompare (version 2.10.1, https://www.danielgm.net/cc/). The 3D data were acquired using the Leica ScanStation in the field. (**c**) Line drawings of *N. ulsanensis* holotype trackway using Adobe Illustrator CC (version 24.0.1, https://www.adobe.com/kr/products/illustrator.html). The dotted areas indicate damaged surface.

Ichnogenus ***Novapes*** ichnogen. nov. (Figs. 2, 3).

**Type species.** *Novapes ulsanensis* ichnogen. et ichnosp. nov., by monotypy and designation herein.

**Diagnosis**. Quadrupedal tracks with a pronounced heteropody; pentadactyl manus impression with claw marks and semi-symmetrical outline, wider than longer; divergence between digit I and V imprints ranges 180° to 210°; the digit IV imprint slightly longer than digit II; entaxonic; pentadactyl pes impression with claw marks and asymmetrical outline (i.e., lateral digits are more developed), longer than wide; webbing between the proximal portion of slender digits; the subequal digits III and IV imprints longer than others (the digit I imprint only 30% in length of the digit IV imprint); the sole pad impression is elongate with a U-shaped “heel”; entaxonic.

**Etymology**. From “*nova*” (Latin) meaning new and “*pes*” (Latin) meaning foot.

**Type locality and horizon**. Daegu Formation, Lower Cretaceous (Albian), an exposed river bottom outcrop (250 m^2^) next to Petroglyphs of Bangudae Terrace (National Treasure No. 285) at Bangudae locality (Ulsan Cultural Property No. 13) in Daegok-ri, Ulju-gun, Ulsan Metropolitan City, South Gyeongsang Province, South Korea.

**Age**. The Daegu Formation was correlated to the Haman Formation based on the bulk composition of lithology, which is known as the Albian based on radiometric dating of the overlying Hakbong basalt and underlying formations^10,11^. Thus, the Daegu Formation is considered Albian in age.

***Novapes ulsanensis*** ichnosp. nov. (Figs. 2, 3).

**Diagnosis**. As for ichnogenus.

**Etymology**. Named after Ulsan Metropolitan City that yielded the holotype.

**Holotype**. A quadrupedal trackway consisting of nine manus-pes sets on a mudstone block (160 × 40 cm, NHCG-a10952: National Heritage Center Geology, Cultural Heritage Administration).

**Age**. Early Cretaceous (Albian).

**Figure 4:**
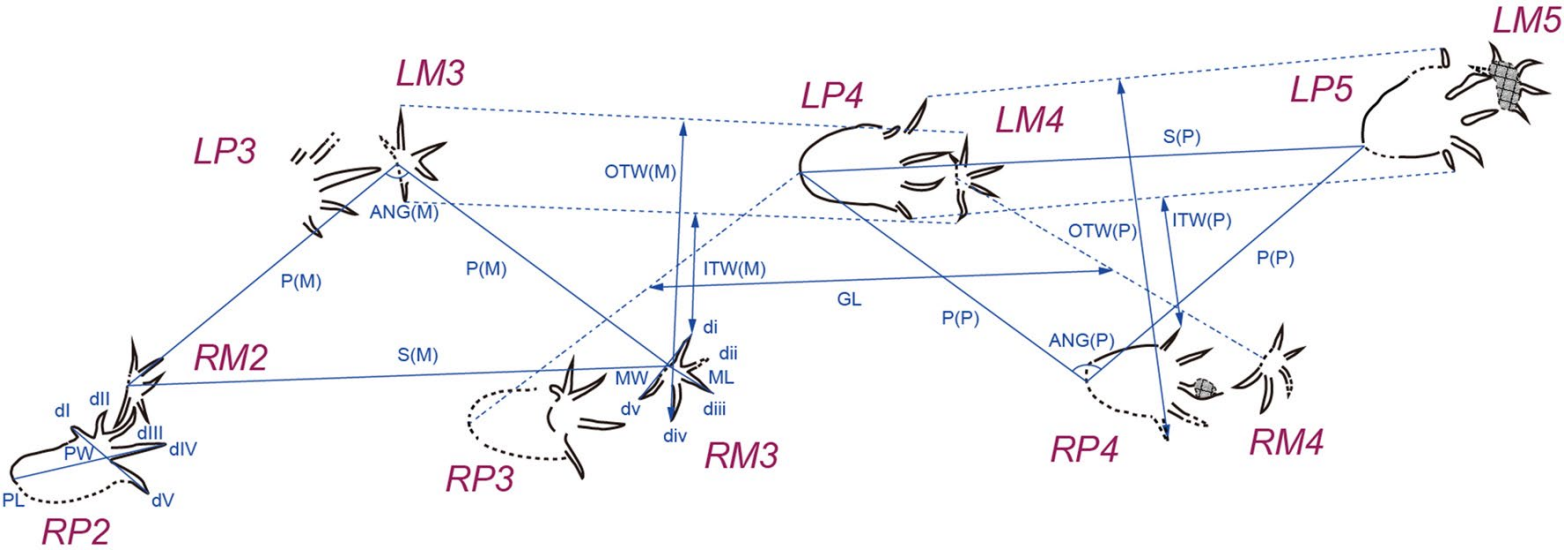
Measurements of *Novapes ulsanensis* footprints and trackway on line drawings using Adobe Illustrator CC (version 24.0.1, https://www.adobe.com/kr/products/illustrator.html). ANG, pace angulation; di ~ dv, manual digit numbers; dI ~ dV, pedal digit numbers; GL, glenoacetabular distance; ITW, trackway width (inner); ML, manus length; MW, manus width; (M/P), manus/pes; OTW, trackway width (outer); P, pace length; PL, pes length; PW, pes width; S, stride length.

**Figure 5:**
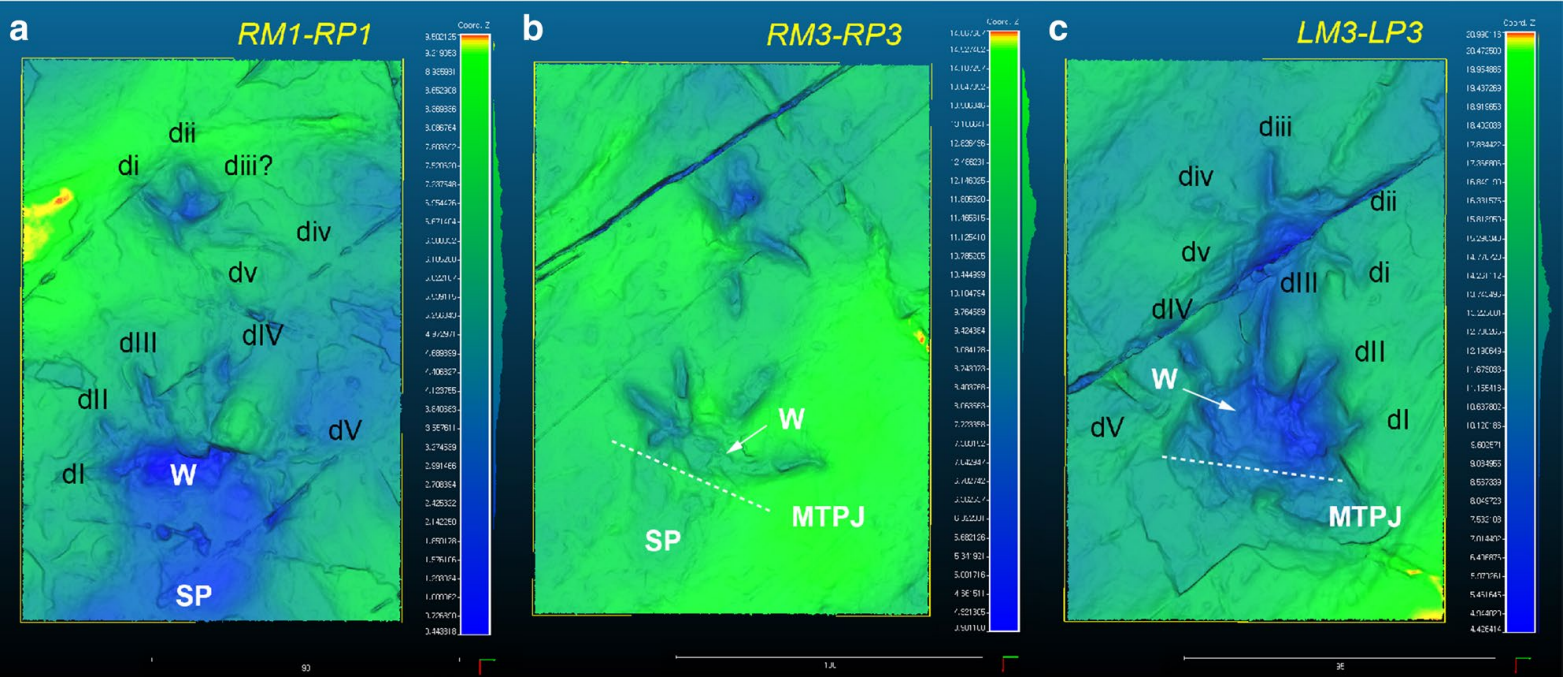
Close-up colour depth map of RM1-RP1, RM3-RP3, and LM3-LP3 with the relative depth legend in meters using CloudCompare (version 2.10.1, https://www.danielgm.net/cc/). The 3D data were acquired using the Konica-Minolta Vivid9i in the field. di ~ dv, manual digit numbers; dI ~ dV, pedal digit numbers; MTPJ, metatarsophalangeal joint line; SP, sole pad impression; W, webbing impression.

**Description**. *Novapes ulsanensis* consists of nine manus-pes sets (5 left and 4 right), indicating quadrupedal locomotion (Fig. 3). The tracks are preserved as positive depressions, appearing true tracks with well-defined impressions of manual and pedal digits and sharp claw marks^12^ (Fig. 2) and tail traces (Fig. 3c). The manus tracks are pentadactyl and plantigrade with the semi-symmetrical outline. The five-digital imprints are terminated with claw marks. The digit impressions are separated from a small and centrally positioned sole pad (Fig. 4). The acropodial portion is deeper than metapodial and basipodial ones. The average length and width are 29.4 mm and 53.0 mm, respectively. The slender digit imprints are straight or slightly recurved. The digit IV imprint is slightly longer than digit II imprint. The digit I imprint is the shortest among manual digit imprints. In the right manus tracks, the digit III imprints are not well preserved, which makes the track appear tetradactyl (Fig. 5a,b). The divergence between digit imprints I-V is between 180°-210°. There is no webbing between digits. In manus tracks, the medial digit imprints tend to be more deeply impressed than lateral ones, likely indicating functional entaxony (Figs. 2a, 5a). The average stride and pace lengths are 349.7 mm and 208.1 mm, respectively. The pace angulation is 119.5° on average (Table 1).

**Table 1:** Measurements (in mm and degrees) of *Novapes ulsanensis*.

Track #	L or R	M or P	L	W	P	S	ANG	ITW	OTW	GL
1	L1	M	–	–	197	300				
		P	–	–						
2	R1	M	31.9	52.4	207	352			238	
		P	–	70.1						
3	L2	M	–	–	233	409	107		143	
		P	–	–				101	213	
4	R2	M	31.8	54.6	196	334	143	69	175	206
		P	107	74	212	275		85	218	
5	L3	M	24.1	54.9	203	347	114	95	205	236
		P	–	61.7	207	339	82	78	219	
6	R3	M	29.5	56.9	204	362	116	63	166	239
		P	97.9	71.6	259	364	93	76	212	
7	L4	M	33.7	51.3	203	344	126	75	181	234
		P	93.1	74	215	347	99	83	214	
8	R4	M	25.5	52.6	222		111	113	210	
		P	105.7	60.3	245		97			
9	L5	M	29.5	48.1						
		P	90.5	73.1						
Average		M	29.4	53.0	208.1	349.7	119.5	83.0	188.3	228.8
		P	98.8	69.3	227.6	331.3	92.8	84.6	215.2	

ANG, pace angulation; GL, glenoacetabular distance; ITW, trackway width (inner); L, footprint length; L/R, left/right; M/P, manus/pes; OTW, trackway width (outer); P, pace length; S, stride length; Track #, track number; W, footprint width.

The pes tracks are oval and fully plantigrade (Fig. 2). They are pentadactyl with anterior claw marks. The slender digit impressions are well separated in the distal portion but covered with webbing about one third from the proximal base as seen in Fig. 5. Digit III and IV imprints are much longer than digit II imprint, while digit I imprint is the shortest. On the whole, the footprint appears distinctly asymmetrical. In particular, the digit I imprint is very short in length compared with other imprints (RP1, RP2, RP3, LP3), which has only 30% of the digit IV imprint on average. The divergence between digit imprints I-V varies among pes imprints but not over 180°. The sole pad impression is elongate (metatarsals) and almost symmetrical to the central axis of the foot as seen in LP4. It ends with a U-shaped “heel” (tarsals) impression posteriorly. The average length and width of pes footprints are 98.8 mm and 69.3 mm, respectively. As manus tracks, the acropodial portion is deeper than metapodial and basipodial ones in pes tracks. The medial portion tends to be more deeply impressed than lateral one, being entaxonic (Figs. 2b, 5a,b). The average stride and pace lengths are 331.3 mm and 227.6 mm, respectively. The pace angulation is 92.8° on average (Table 1).

**Figure 6:**
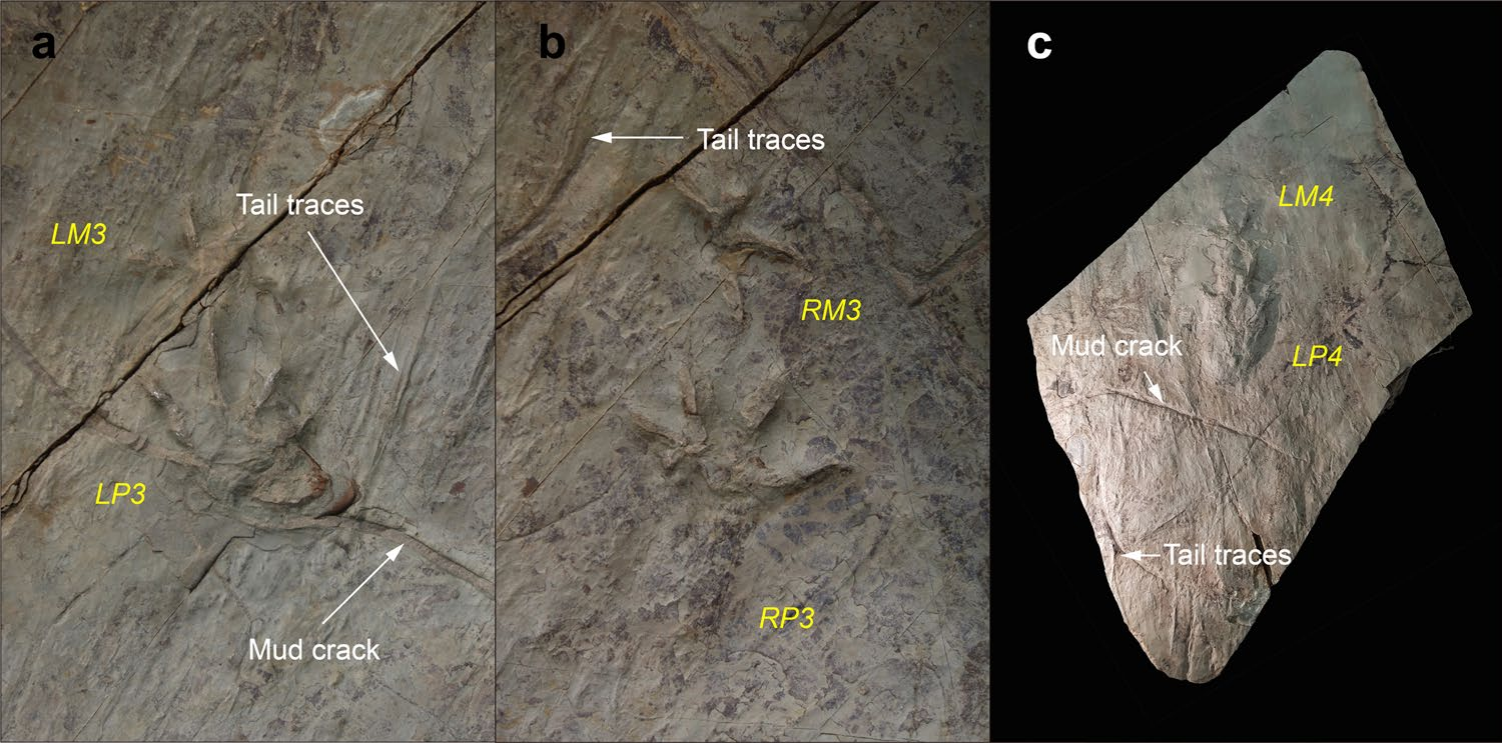
Close-up photos to show tail traces and mud cracks. (**a**) Tail traces on the right side of Track number 5 (LM3-LP3). (**b**) Tail traces on the left side of Track number 6 (RM3-RP3). (**c**) A block containing natural casts of Track number 7 (LM4-LP4) in convex hyporelief with tail traces and mud cracks.

The trackway is characterized by strong heteropody (ratio of manus to pes imprint length 1:3.36 and width 1:1.3). The trackway width is about three times the pes width. All manus tracks are closely located in front of pes tracks and their trackway width is narrower than that of pes (average 188.3 mm and 215.2 mm in the outer trackway width). The axis of the manus prints (here traced parallel to the direction of the digital III imprint) is directed much more anterolaterally than that of the pes prints (here traced parallel to the direction of the axis of sole pad impression), showing strong positive rotation (Fig. 5b). The rotation angle is much larger in the right manus (about 50°) than left one (15°). The left manus are positioned lateral to or in front of the pes while the right manus are medial to the pes. The trackway curved slightly towards the right at the beginning and went straight to the south. There is no belly mark but tail traces (Figs. 3c, 6). Tail traces occurs as fine striations between left and right footprints, which are slightly sinuous to nearly linear. However, the tail drag marks are neither symmetrical nor placed close to the trackway midline.

**Remarks**. Pentadactyl pes tracks are easily distinguished from tetradactyl pes tracks of crocodyliform ichnotaxa such as *Crocodylopodus*^13^ and *Batrachopus*^14^. Divergence of digit imprints I-IV is 20° to 55° in crocodyliform pes imprints^15^ while the divergence reaches almost 180° between digit imprints I-V of *N. ulsanensis* (RP2, RP3). Similar pentadactyl pes tracks are present in the non-pterodactyloid pterosaurian *Rhamphichnus*^16^, but it presents tridactyl manus tracks. Turtles typically have short and symmetric pentadactyl manus and pes imprints. However, they never show a pronounced heteropody^6^. Their tracks are always nearly in parallel rows due to the constraints imposed by the presence of the shell^17^. Besides, amphibians have pentadactyl pes with rounded tips of digits, generally without claw marks^18^. Interestingly, “typical” lizard trackways are characterized by strong heteropody with pentadactyl manus and pes imprints such as *Sauripes*^19^ and *Neosauroides*^20,21^. However, even lizards that swim do not have webbing between toes^22^. In addition, digit V imprint is distinctly separated from other imprints and is connected to the back of the heel trace^19^.

Notably, *Champsosaurichnus parfeti* was named based on two isolated tracks found in the Upper Cretaceous Laramie Formation of Colorado, USA^23^. The authors admitted that it is uncertain if the holotype is a manus or pes, or if the two tracks really represent a step or a stride. Nevertheless, the authors alleged them the first champsosaur ichnofossils on the basis of skeletal (compared with *Champsosaurus laramiensis*, Neochoristodera) and ichnological similarities that include overall symmetry, digits II and IV about 75% of the length of digit III being subequal in length. However, the fore- and hind- foot of *Champsosaurus laramiensis* is not symmetrical outline and the digit IV is subequal to the digit III rather than the digit II^24^. *Champsosaurichnus parfeti* is also quite different from *N. ulsanensis* in having a small transverse heel of pes imprint and no interdigital web traces. Therefore, *N. ulsanensis* is neither matched with any web-footed tracks reported before nor with *Champsosaurichnus parfeti*.

## Discussion

The well-preserved interdigital web traces of pes imprints clearly show that *N. ulsanensis* was left by a web-footed animal with five functional digits. The prominent one-half webbing (a semi-palmate) impressions occur in between all digits (Fig. 5). In particular, LP3 print shows the deeply impressed digits II, III, and IV associated with a proximally impressed area anteriorly bordered by transversely straight outlines between them while the heel impression is shallow (Fig. 5c). It indicates that it was made when the bodyweight of an animal was transferred to the acropodium during the kick-off phase, thereby having a high possibility to leave real web traces. They seem not to be an interdigital structure formed by through sediment failure^25^ because they are clearly developed only within pedal geometry and impressed as deep as the level of digit impressions. If they are sediment displacements, a similar structure should have occurred in the manus impressions, but there is none.

Choristoderans are freshwater aquatic or semi-aquatic diapsid reptiles that lived from the Middle Jurassic through to the Miocene^26,27^. They are often compared to crocodilians, occupying similar niches as a good example of convergent evolution^28^. While abundant crocodyliform tracks have been reported in the Jurassic and Cretaceous in the world^8,29^, however, it is a bizarre phenomenon that there is no single report thus far on the choristoderan tracks except for *Champsosaurichnus parfeti*. It is probably because some of them were exceptionally well adapted to life in the water such as long-necked genera *Shokawa*^30^ and *Hyphalosaurus*^31^. However, some genera could have been more terrestrial due to the nesting behaviour on land^32^. For instance, *Monjurosuchus* was able to go onshore to lay eggs or attack the nests to eat young individuals^33^. Interestingly, choristoderans had five manual and five pedal digits with sharp claws and their fossil occurrences show the highest diversity in the Early Cretaceous of eastern Asia, especially in China as well as in Japan and Mongolia^34,35^. It suggests that these regions including Korea were an important centre for choristoderan evolution because choristoderes were more tolerable in low temperature than crocodyliforms during the Early Cretaceous^36,37^.

Although seven choristoderan genera were reported in the Early Cretaceous of Asia, well-preserved hands and feet are known in *Hyphalosaurus* and *Monjurosuchus*^31,38^. While long-necked and fully aquatic *Hyphalosaurus* inhabited exclusively in deep-water lakes, short-necked and semi-aquatic *Monjurosuchus* lived in a more shallow-water ecosystem^31^. The soft tissue traces on *Monjurosuchus* show that it had webbed toes on both manus and pes with only the claws projecting^38^. In contrast, there is no indication of interdigital webbing in the manus of *N. ulsanensis*. However, a Paleocene choristoderan *Lazarussuchus* resembling *Monjurosuchus* in body size and shape, that preserves traces of soft tissue, does not show webbing^39^. There are also observable differences in the extent of interdigital pedal webbing in the extant crocodilian species^40^. It is reasonable, therefore, to assume that there were the morphological variations of interdigital webbing in manus and pes among choristoderan taxa. Except for the absence of interdigital webbing in the manus, *N. ulsanensis* is fairly well matched with foot skeletons of *Monjurosuchus* such as the hindfoot much larger than the forefoot, phalangeal length greater than the length of metatarsals based on the position of metatarsophalangeal joint lines, the shortest pedal digit I with similar length of digits III and IV which are longer than digit II, and webbed hind feet (Figs. 2, 5). Notably, the manus trackway width is narrower than that of pes in *N. ulsanensis*, maybe reflecting the forelimbs are 75% shorter than hind limbs and the distance between left and right shoulder joints is shorter than that between hip joints of *Monjurosuchus*^38^. On the other hand, pes prints of crocodiles are usually positioned just behind ipsilateral manus prints of the same set^41^. In addition, the average of glenoacetabular length of the trackway is 228.8 mm, which is concordant with the body size of *Monjurosuchus* whose snout-vent length [SVL] is up to 300 mm. Therefore, *Novapes ulsanensis* could be made by a monjurosuchid-like choristoderan.

Most of choristoderans were primarily aquatic, exceptionally well adapted to life in the freshwater. Female individuals of *Champsosaurus*, however, were believed to be better adapted to terrestrial life due to their nesting behaviour on land, on the basis of limb morphological and histological differences between males and females^32,42,43^. The differential depth of the impression can be used for evaluation on biomechanics and functionality of the trackmaker in the light of osteological structure^44–46^. The functional axis in manus imprints of *N. ulsanensis* presents that the forelimbs of a trackmaker moved always along the medial side (entaxonic) during the stroke progression (Figs. 2, 5), while the functional axes in pes imprints indicate that the hind limbs moved first centrally (mexasonic) during the touch-down phase and moved medially (entaxonic) during the maximum load phase and kick-off phase (Fig. 5), allowing for the inference of a locomotion cycle of choristoderans.

**Figure 7:**
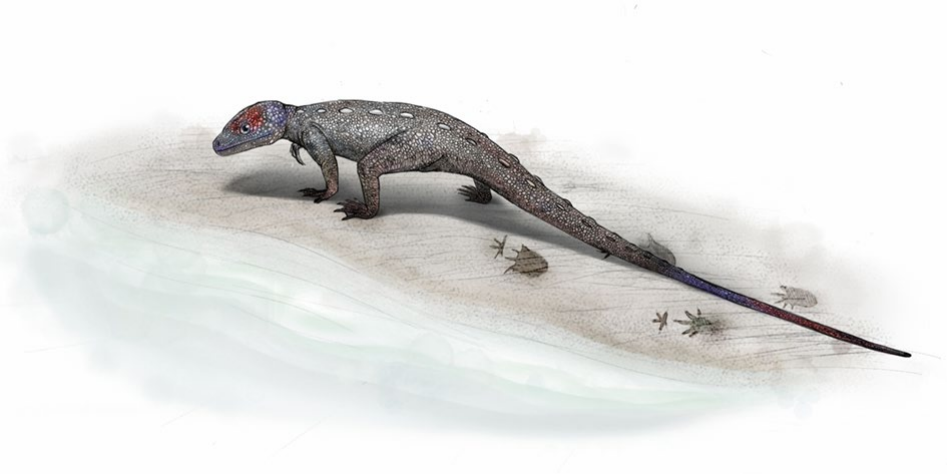
A reconstruction of a *Monjurosuchus*-like choristoderan walking quadrupedally on the lakeshore leaving *Novapes ulsanensis* behind (Drawn by Ui Dong Jung).

Modern crocodilians show two locomotory postures, namely ‘high-walk (semi-erect)’ and ‘low walk (sprawl)’^47^. They can slide on its belly in the soft or slippery substrate, using a sprawling posture. This mode of locomotion leaves wide belly mark with curved drag marks^15,40^. The high walk is used for overland travel and the body is held off the ground. Kubo and Ozaki^48^ demonstrated that a high walk posture produces a trackway with high pace angulation and concluded that a trackway with an average pace angulation value of 108° or less is unlikely to have been produced by an animal with a fully erect gait. The pes pace angulation of *Crocodylus porosus* varies between 92° and 115° in high walk posture while it varies between 73° and 76° in sprawling^15^. The pes pace angulation value of *N. ulsanensis* ranges from 82° to 99° (92.8° on average, Table 1), which is more concordant with the crocodilian ‘high walk’ than the ‘sprawling’ posture. The aquatic or semi-aquatic choristoderans had a long tail which played the major role in swimming^38^ but constituted a source of drag when the animal walks on land as shown in *N. ulsanensis* (Figs. 2c, 6). In addition, the manus prints rotate distinctly outward while the pes prints are nearly parallel to the direction of travel in *N. ulsanensis* as seen in the trackways of the American crocodile (*Crocodylus acutus*)^41^. Interestingly, the position and larger rotation of right manus than left one with the stride LM2 longer than the respective on the right side is compatible with a producer turning toward the right. Therefore, *N. ulsanensis* implies that a *Monjurosuchus*-like choristoderan was actually able to use the semi-erect, high walk posture with tail-dragging when moving on the ground like crocodilians, further supporting the choristoderan adaption to shallow freshwater habitats (Fig. 7).

## Methods

### Geological setting

The tracksite occurs in the Daegu Formation distributed in the southeastern of the Gyeongsang Basin^49^ (Fig. 1a). In general, the Daegu Formation consists of reddish sandy siltstones, siltstones, sandy shales, shales, and greenish or dark grey sandstones. The formation is well exposed along the cliff and river bottom of the Daegok River. This area contains at least 20 vertebrate ichnofossil-bearing horizons, which produced approximately 80 ornithopod, sauropod, theropod, and pterosaur tracks^50^. Various sedimentary structures such as mud cracks, ripple marks, and raindrop prints occur on the surface with invertebrate trace fossils, indicating that this formation was deposited under semi-arid paleoclimatic conditions with strong seasonality^51^. This interpretation is supported by the extensive development of dry to saline mudflat deposits associated with common evaporate mineral casts, complicated polygonal desiccation cracks and raindrop prints, and pedogenic carbonate development^52^. The flora of conifer woods also supported semi-arid paleoclimatic condition during the Cretaceous in the Korean peninsula^53^. The new footprint site (35°36.255′N, 129°10.706′E) was discovered in 2018 during the surface prospecting in the vicinity of Petroglyphs of Bangudae Terrace (National Treasure No. 285) of which the lower strata are supposed to be submerged underwater after building a dam in near future. The explored area is about 250 m^2^ (25 m × 10 m) which contains seven track-bearing horizons in 14 m thickness (Fig. 1b–d). Most of the footprints occur in the lower part which are ornithopod and bird trackways as well as a new quadrupedal trackway of this study. The development of the fine-grained cyclic deposits commonly associated with mud cracks at the site indicates the lake shoreline paleoenvironments. The track (*N. ulsanensis*) bearing horizon has neither ripple marks nor burrows, but complicated polygonal desiccation cracks (Fig. 6), suggesting the subaerial exposure. After 3D scanning in the field, this new trackway (nine sets of quadrupedal tracks, 160 cm in length) was excavated and stored in the National Heritage Center, Daejeon City.

### Methodology and terminology

Track and trackway data were systematically measured (Fig. 4) following crocodilian ichnological terminology^41^ in general and the parameters of each track are given in Table 1. They measured manus print length in a straight-line distance from the terminus of the digit III impression to the proximal edge of the manus print but its width from the terminus ends of the impressions of digits I and V rather than perpendicular to manus print length (Leonardi protocol)^5^. Pes print length was measured from the most posterior point on the heel impression to the tip of the longest toe (digit IV) while its width measured across the terminal ends of the impression of digits I and V. Manus pace was measured from the most posterior point on the wrist impression (i.e., the manus reference point) rather than the terminus of the digit III impression to the same point on the next manus print of the opposite side because of the variability of digit III position while pes pace from the most posterior point on the heel impression (i.e., the pes reference point) to the same point on the next pes print of the opposite side. Manus stride was measured from the most posterior point on the wrist impression to that of the next one of the same side while pes stride from the most posterior point on the heel impression to that of the next one of the same side. Manus and pes pace angulations are the angle of two successive manus and pes paces, respectively. Trackway inner (internal) width is a series of line segments that connects the most medial part of manus and pes prints (the terminal end of the digit I impression) for the manus and pes trackway, respectively. On the other hand, Leonardi^5^ measured it “between the internal parallel tangents to two consecutive left–right footprints.” Trackway outer (external) width is a series of line segments that connects the most lateral part of manus and pes prints (the terminal end of the digit V impression) for the manus and pes trackway, respectively. Glenoacetabular length is the length of a line segment between the midpoint of a line segment connecting the reference point of two successive contralateral pes prints and the midpoint of a line segment connecting the reference point of two successive contralateral manus prints. The term ‘axony’ is used for the maximum load phase to define footprints, thus indicating a corresponding functional axis^5,54^. The term ‘heteropody’ is used for the condition in which the hand and the foot are dimensionally and morphologically different^5^.
